# Effects of Reactive Oxygen Species on *in vitro* Filtration of Water and Albumin across Glomerular Basement Membrane

**Published:** 2006-06

**Authors:** Ehab I. Mohamed, Naglaa M. Fahmi, Soher M. El Kholy, Samera M. Sallam

**Affiliations:** 1 *Division of Human Physiology, Faculty of Medicine and Surgery, University of Tor Vergata, Rome, Italy;*; 2 *Department of Biophysics, Medical Research Institute, University of Alexandria, Alexandria, Egypt;*; 3 *Department of Physics, Faculty of Science, University of Alexandria, Alexandria, Egypt;*; 4 *Department of Physics, Faculty of Science, University of Benha, Benha, Egypt*

**Keywords:** reactive oxygen species, glomerular basement membrane, ultrafiltration cell, darcy permeability, albumin flux, sieving coefficient

## Abstract

Most of the interest in the glomerular basement membrane (GBM) stems from the observation that it undergoes morphological changes in renal disease. Studies on persistent proteinuria in experimental animal models have shown that the permeability properties of the GBM have been altered as a result of protein degradation and cross-linking of type IV collagen via its NC1 domains promoted by reactive oxygen species (ROS) and extrusion of tubular cell contents. We used the *in vitro* ultrafiltration technique to assess permeability properties of bare isolated GBM films to water and albumin in the Munich Wistar Fromter rat model of glomerular injury. Hydraulic permeability for water and albumin solutions and albumin fractional clearances were measured for rats treated with lisinopril [an angiotensin converting enzyme (ACE) inhibitor] and were compared with those measured for rats treated with dimethylthiouria (an ROS scavenger) and their control groups, at four pressure levels (50, 100, 200, and 300 mmHg). The ACE inhibitors and ROS scavengers treatment regimens for studied rats in addition to significantly lowering their systolic blood pressure and urinary protein excretion values to normal levels, have significantly increased their *in vitro* hydraulic and Darcy permeability, which is a measure of the intrinsic hydraulic conductance of the GBM, either in the absence or presence of albumin; in comparison with control animals. We believe that these favorable effects may derive from ROS scavenging beneficial effects that preserve the GBM protein structure by reducing entactin and laminin degradation and type IV collagen cross-linking.

## INTRODUCTION

Proteinuria has been considered as an independent risk factor for the progression of renal disease, which has been frequently associated with an increasing vascular permeability to circulating proteins (e.g., albumin and IgG), experimentally observed to accumulate in the cytoplasm of proximal tubules (1). Proteinuria has also been found to be associated with a decreasing glomerular filtration rate due to the loss of either size- or charge-selective properties of the glomerular barrier (1, 2). The glomerular capillary wall (GCW) consists of the glomerular basement membrane (GBM), which is situated between highly differentiated endothelial and epithelial cell layers. The three layers act together to significantly retard the flux of macromolecules, while allowing a very large water flux. However, the contribution of each layer to the overall permselective properties of the GCW has been difficult to determine (3-5). The GBM has been considered to be the only continuous barrier, which prevents blood and various circulating proteins from passing into the tubular lumen, and its thickness is approximately twice that of the basement membranes of other tissues (3). A recent modeling study based on an ideal distribution of GBM proteins assembled in an uniform three-dimensional meshwork has shown that the GBM was the principal determinant for the permselective function of the GCW (6).

In persistent proteinuria, studies using experimental models have shown that the permeability properties of the GBM are altered as a result of loss of integrity, which could be caused by either the cross-linking of the NC1 domains of type IV collagen molecules, promoted by reactive oxygen species (ROS) (7, 8), or by the extrusion of tubular cell contents, which causes inflammation and fibrosis (9-11). The ROS (e.g., superoxide, hydrogen peroxide, hydroxyl radical, and hypochlorous acid) are generated in copious amounts by the intact kidney, reflecting the relatively high rates of oxygen consumption that characterize renal metabolism (12). The ROS may be generated in the glomerular capillaries by the infiltrating blood cells, which are activated upon contact with the immune complexes (13, 14), the GBM (15), or the glomerular mesangial cells (16, 17). It has been shown that ROS play a pathobiologic role in a number of experimental models of both immune and non-immune glomerular injury (18), mediating renal damage in acute renal failure and glomerular and tubulointerstitial diseases (19).

ROS oxidize sensitive sulfhydryls on proteins, peroxidate lipids in membranes, depolymerize polysaccharides, and degrade nucleotides or nucleic acids, which may explain the increased excretion of GBM fragments in the urine in a variety of human and experimental glomerular diseases (2). When ROS are produced in the glomerular circulation, the glomerular filtration rate falls due to renal vasoconstriction and to decreases in the glomerular capillary ultrafiltration coefficient (20). It has been shown that treatment with dimethylthiouria (DMTU) reduces urinary protein and albumin excretion and increases the glomerular filtration rate in experimental animal models (21, 22).

The objective of the present study was to investigate the *in vitro* permeability properties of bare isolated GBM to water and macromolecules independently from glomerular hemodynamics and glomerular cells, using an ultrafiltration cell in the experimental model of glomerular injury of male Munich Wistar Frömter (MWF) rats. Based on this, we studied whether the renoprotective effects of angiotensin converting enzyme (ACE) inhibitors and ROS scavengers, which have been observed in these animals *in vivo*, derive from modulations of GBM hydraulic and macromolecular permeabilities.

## MATERIALS AND METHODS

### Study Design

The present study consisted of 30 male MWF rats which were bred at the Mario Negri Institute for Pharmacological Research (Bergamo, Italy). The rats had continuous free access to food (standard rat chow containing 20% protein by weight) and tap water. Beginning at 10 weeks of age, 8 rats received lisinopril, an angiotensin converting enzyme (ACE) inhibitor (Zeneca Pharmaceutical, Macclesfield, UK) at a dose of 40 mg·L^-1^ in drinking water for an average period of 18 weeks. These rats are referred to as the “LIS” group (mean age at sacrifice = 27.15 ± 1.10 weeks). As controls, we used 7 rats that received no pharmacological treatment and that were observed until reaching an average age of 28.80 ± 2.72 weeks (group referred to as “CTR1”). Eight rats, beginning at 10 weeks of age, received dimethylthiourea, an ROS scavenger (Aldrich Chem. Co., WI, USA), at a dose of 250 mg·kg^-1^ body weight, dissolved in saline and administered as a single daily intraperitoneal injection, for an average period of 6 weeks (“DMTU” group; mean age at sacrifice = 17.71 ± 0.52 weeks). As the second control group (CTR2), 7 rats received no pharmacological treatment and were observed until reaching an average age of 18.62 ± 0.67 weeks.

During the observation period, systolic blood pressure (SBP) was measured once a week by tail plethysmography in awake rats (23). The rate of urinary protein excretion (UPE) was determined once a week by 24-hour urine collection in metabolic cages, and protein concentrations in urine were determined using the Coomassie blue G dye-binding assay, as previously described (24). Both the procedures for animal care and the applied experiments were conducted in accordance with the institutional guidelines outlined by national and international laws and policies (ECC Council Directive 86/609, OJL 358-1, NIH Guide for the Care and Use of Laboratory Animals).

### Isolation of the GBM

Adult male MWF rats (230-420 g) were anesthetized with inactin (100 mg·kg^-1^, intraperitoneally), and to remove blood both kidneys were perfused *in situ* at 100-150 mmHg with Tris-buffered saline (0.15 M NaCl, 0.05 M Tris-hydroxymethyl aminomethane/HCl buffer, pH7.4). Perfusion pressure was monitored using a high sensitivity pressure transducer (Battaglia Rangoni, Bologna, Italy). The kidneys blanched promptly and the perfusion was completed within 2 min.; the kidneys were then removed and weighed. All subsequent steps were performed at 4°C. The renal capsules, extrarenal vessels, and papillae were discarded, and the cortexes were separated from the medulla, weighed, and minced. The resulting homogenate was sequentially passed through standard nylon sieves (pore size of 180 and 150 μm) (Gioliani, Torino, Italy), which excluded most of the tubules, and washed in Tris-buffered saline (pH7.4) containing 0.1% Tween over a 75 μm sieve to keep glomeruli. Microscopic quantification showed that the resulting glomerular preparation contained less than 5% tubular fragments and over 95% decapsulated glomeruli.

Glomeruli were subjected to detergent lysis with N-lauryl-sarcosine (Sigma, St. Louis, Mo., USA), as previously described (25). Briefly, 20 ml·g^-1^ cortex N-lauryl-sarcosine (0.5% wt/vol in Tris-buffered saline) was added to the glomerular pellet and vigorously homogenized for 2 min. After standing for 10 min., the suspension was centrifuged at 2000 × g for 2 min., and the sediment was resuspended in fresh detergent (4 ml·g^-1^ cortex), vigorously shaked, and centrifuged at 2000 × g for 2 min. The residue was washed once in Tris-buffered saline, suspended in unbuffered 0.15 M NaCl containing 0.01% (wt/vol) deoxyribonuclease-1 (1.5 ml·g^-1^ cortex, Type DN 25; Sigma Chemical Co., St. Louis, MO), vortexed, and allowed to stand for 30 min. at room temperature. The glomeruli were then centrifuged, suspended in 4 ml of Krebs buffer (120 mM NaCl, 4.8 mM KCl, 1 mM KH_2_PO_4_, 1.2 mM CaCl_2_, 0.6 mM MgSO_4_, 24 mM NaHCO_3_, 180 mg·dL^-1^ glucose; pH adjusted to 7.4 before use), and sonicated using an ultrasonic cell disrupter (Microson, Heat Systems Inc., NY, USA) for 1 min. to disrupt what may have remained of glomerular capsules. Membrane fragments were then used for filtration experiments. All solutions were filtrated (0.22 μm pore size filters; Millipore, Bedford, MA, USA) immediately before use to remove contaminants, and glassware was precoated with silicon (Sigmacote; Sigma Chemical Co., St. Louis, Mo, USA) to minimize the sticking of glomeruli or GBM.

### Ultrafiltration Cell

A mini-ultrafiltration cell (Type 3, Amicon Inc., Beverly, MA, USA) was modified to include a sampling port for periodic sampling of the retentate and buffer refilling, which was connected to the N_2_ pressure line through a roller pump (Venous Line Pressure, Bellco, Italy) via a three-way stopcock. A Whatman 50 hardened filter paper (Whatman International Ltd., Springfield Mill, UK) and an HAWP 0.45 μm Millipore polysulfone filter (Millipore, Ireland), both placed at the base of the ultrafiltration cell, were used as supporting filters. The packing pressure and the pressure inside the cell during filtration experiments were monitored using a pressure transducer (World Precision Instruments Inc., Sarasota, FL, USA), which was connected to the stopcock. The ultrafiltration cell was placed on top of a calibrated magnetic stirrer (FALC, Disa, Milan, Italy).

### Filtration Experiments

The GBM suspension (150 μg in Krebs buffer) was loaded in the cell, and the pressure inside the cell was gradually increased to 1.5 atm. and maintained for 1 hour to consolidate the GBM into a homogenous layer resting on the polysulfone filter. The effect of the difference in transmembrane hydraulic pressure on the hydraulic permeability of the GMB was studied by filtering buffer solutions at the following discrete pressures: 50, 100, 200, and 300 mmHg. At each pressure level, an equilibration period of 3 min. followed by a collection period of 5 min. in pre-weighed test tubes were applied. The buffer was removed and the cell was carefully rinsed with 3 ml of retentate solution containing albumin (4 g·dL^-1^ in Krebs buffer) and then assembled with the magnetic stirrer. Albumin filtration was first performed at a pressure of 300 mmHg and under continuous stirring at 220 rpm for 20 min. for equilibration, followed by a 10 min. collection period. The pressure was subsequently decreased to 200, 100, and 50 mmHg, and the clearance of albumin was determined for each pressure level after a 10 min. equilibration period. The retentate was sampled at the beginning and at the end of each collection period, and the filtrate was collected throughout the 10 min. clearance in pre-weighed tubes. The flux of water and albumin solutions and the clearance and the fractional clearance of albumin were calculated. The albumin concentration was determined in both filtrate and retentate fractions by Coomassie blue G dye-binding assay (24). All filtration experiments were carried out at 25°C. At the end of each filtration experiment, the GBM-polysulfone filter was perfused with Krebs buffer at 50 mmHg for 10 min., fixed in gluteraldehyde 2.5% at the same pressure for 20 min., and finally embedded, sectioned, and stained with periodic acid-Schiff for light microscopy and morphometric studies.

### Calculations

The volumetric flow rate (Q_a_) was estimated by collecting the filtrate for a definite period of time in calibrated test tubes and was used to calculate the volume flux (J_v_) using the formula:

[Eq. 1] $$J_{v}=Q_{a}/A\left({\mathrm{cm}.\sec}^{-1}\right)$$

where A is the membrane effective surface area available for filtration (= 1.54 cm^2^ in our experiments). The filtrate hydraulic permeability (L_p_) was calculated with the formula:

[Eq. 2] $$L_{p}=J_{v}/\Delta P\left({\mathrm{cm}\cdot \sec}^{-1}\cdot {\mathrm{mmHg}}^{-1}\right)$$

where ΔP is the difference in transmembrane hydraulic pressure. When albumin is the only osmotically active solute (as in this study), the hydraulic permeability for albumin solutions (L_p,alb_) is related to J_v,alb_ using the formula (26):

[Eq. 3] $$L_{p,\mathrm{alb}}=J_{v,\mathrm{alb}}/\left({\Delta P-\sigma \mathrm{alb}\cdot \Delta \prod }_{\mathrm{alb}}\right)\left({\mathrm{cm}\cdot \sec}^{-1}\cdot {\mathrm{mmHg}}^{-1}\right)$$

where σalb is the albumin reflection coefficient and ΔΠ_alb_ is the difference in osmotic pressure for albumin (ΔP >> ΔΠ_alb_ in this study). The transport of albumin through the GBM layers was assumed to occur primarily by convection rather than by diffusion (27), thus, σ_alb_ = 1 - Θ_alb_, where Θ_alb_ is the membrane sieving coefficient for albumin, which is given by the ratio of albumin concentration in the filtrate to its concentration on the retentate side immediately adjacent to the membrane surface (i.e., C_f_ /C_m_); thus L_p,alb_ is given by:

[Eq. 4] $$L_{p,\mathrm{alb}}=J_{v,\mathrm{alb}}/\left[\Delta P-\left({1-\Theta }_{\mathrm{alb}}\right)\right]\Delta \mathrm{II}\left({\mathrm{cm}\cdot \sec}^{-1}\cdot {\mathrm{mmHg}}^{-1}\right)$$

σ_alb_ was calculated from the albumin concentration (C_alb_) (g·dL^-1^) using the Landis-Pappenheimer equation given by:

[Eq. 5] $${\mathrm{II}}_{\mathrm{alb}}={2.800C}_{\mathrm{alb}}+0.180{\left(C_{\mathrm{alb}}\right)}^{2}+0.012{\left(C_{\mathrm{alb}}\right)}^{3}\left(\mathrm{mmHg}\right)$$

The actual sieving coefficient (Θ_alb_) was calculated from the effectively measured sieving coefficient (Θ’_alb_), which is the ratio of albumin concentration in the filtrate to its concentration in the retentate (i.e., C_f_/C_r_), using the formula corrected for concentration polarization given by (27, 28):

[Eq. 6] $$\Theta_{\mathrm{alb}}={\Theta '}_{\mathrm{alb}/}\left[\left({1-\Theta '}_{\mathrm{alb}}\right){B+\Theta '}_{\mathrm{alb}}\right]$$

According to Eq. 6, Θ_alb_ will always be less than Θ’_alb_, except for the following cases, in which they will be equal: when a solute is filtered freely (i.e., Θ’_alb_ = 1) or perfectly retained (i.e., Θ’_alb_ = 0) or when there is no concentration polarization (i.e., B = 1).

The effect of concentration polarization is that of sedimenting albumin at the base of the cell, which forms a layer of high albumin concentration near the surface of the GBM film (i.e., C_m_>C_r_) (27). Under the conditions used here, we estimate that Cm typically exceeds Cr by ~20%. The concentration polarization factor (B) is calculated by:

[Eq. 7] $$B=\mathrm{Exp}\left(J_{v,\mathrm{alb}}/K_{c}\right)$$

where the mass transfer coefficient (K_c_), which is a function of albumin diffusivity (D), albumin kinematic viscosity, and the rotation speed of the stirrer, was found to be equal to 4.36 × 10^-4^(cm·sec^-1^). Albumin diffusivity (D) was calculated on basis of the Stokes-Einstein equation for the diffusion coefficient (29), given by:

[Eq. 8] $$D=\mathrm{kT}/6\mathrm{II}\eta a_{e}\left({\mathrm{cm}}^{2.}\sec^{-1}\right)$$

where k is Boltzman’s constant, T is room temperature, η is albumin dynamic viscosity and ae is albumin molecule radius. The albumin permeability coefficient (P_s_) is related to D by the formula:

[Eq. 9] $$P_{s}=D/\delta_{\mathrm{bm}}\left({\mathrm{cm}\cdot \sec}^{-1}\right)$$

where δ_bm_ is the basement membrane film thickness (cm). The albumin flux (J_s_) across the GBM film is given by (27):

[Eq. 10] $$J_{s}={\Theta '}_{\mathrm{alb}}\cdot C_{r}\cdot J_{v,\mathrm{alb}}\left(g\cdot {\mathrm{cm}}^{-2.}\sec^{-1}\right)$$

The local membrane Darcy permeability, which is a measure of the intrinsic hydraulic conductance of the GBM film, both in the absence of albumin (K_d_) and in the presence of albumin (K_d,alb_), is given, respectively, by (30):

[Eq. 11a] $$K_{d}=\eta \cdot \delta_{\mathrm{bm}}\cdot L_{p}\left({\mathrm{cm}}^{2}\right)$$

[Eq. 11b] $$K_{d,\mathrm{alb}}=\eta_{\mathrm{alb}}\cdot \delta_{\mathrm{bm}}\cdot L_{p,\mathrm{alb}}\left({\mathrm{cm}}^{2}\right)$$

where η and η_alb_ are the dynamic viscosity (g·cm^-1^·sec^-1^) for the buffer and albumin solutions, respectively, and L_p_ and L_p,alb_ are the actual hydraulic permeability in the absence and presence of albumin, respectively. A glass viscometer was used to measure both η and η_alb_ from direct estimation of their kinematic viscosities at room temperature (25°C).

### Morphometric Measurements

To determine the thickness of GBM films (δ_bm_), we applied standard morphometric techniques (31) to sections of GBM-polysulfone filter using a computer-based image analysis system comprised of an upright light microscope (Carl Zeiss, Oberkochen, Germany) equipped with a video camera (Panasonic, Matsushita Elect. Co., Osaka, Japan) connected to a computer (Macintosh IIFx, Apple Computer Inc., Cupertino, CA, USA) (32). Measurements were taken using the Image software package (NIH-Image, National Institutes of Health, USA). Briefly, serially acquired images of the GBM film sections were randomly oriented and digitally overlaid with an 8 × 9 orthogonal lines reference grid; the GBM film thickness was measured in screen pixels along the grid lines intersecting the membrane vertically and horizontally. The exact enlargement of the acquired GBM images was calculated from the direct measurement of a reference grid with a micrometer eyepiece (Nachet, Paris, France). A minimum of 500 measurements in both directions were applied for each filter. The mean thickness of GBM films (δ_bm_) was calculated using the harmonic mean δ_h_ using the formula:

[Eq. 12] $$\delta_{\mathrm{bm}}=8/3\mathrm{II}\cdot \delta_{h}\left(\mathrm{cm}\right)$$

where the 8/3Π is a correction factor for variations in the sectioning angle through the GBM.

### Statistical Analysis

Data analysis was carried out using the StatView® statistical package (SAS Institute Inc., NC, USA). One-way analysis of variance (ANOVA) and Scheffe’s post-hoc test of significance were applied to compare different variables among different groups. Variables are expressed as mean ± SD, unless otherwise stated. Differences among variables were considered significant at *p*<0.05.

## RESULTS AND DISCUSSION

### Physiologic Data

**Figure 1:**
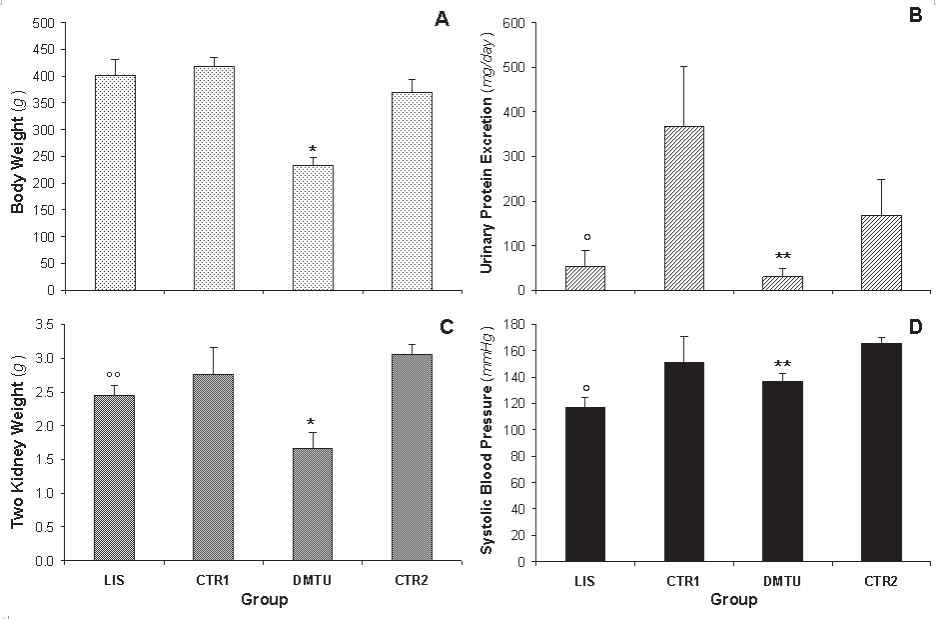
Body and two kidney weight, urinary protein excretion in 24 hours and systolic blood pressure for lisinopril treated rats (LIS, n = 8), its negative control group (CTR1, n = 7), dimethylthiouria treated rats (DMTU, n = 8), and its negative control group (CTR2, n = 7). Data are presented as mean ± SD. *p<0.0001 vs. CTR2, **p<0.05 vs. CTR2, °*p*<0.0001 vs. CTR1, and °°*p*<0.05 vs. CTR1.

The results of the body weight, two-kidney weight, UPE, and SBP are shown in Figure 1. We did not recognize significant differences in body weight when comparing the LIS group to the CTR1 group; however, the average body weight for the DMTU animals was 36.90% (*p*<0.0002) lower than that for the CTR2 group (Figure 1A). The two-kidney weight for the LIS and DMTU groups (2.45 ± 0.15 and 1.66 ± 0.24 g, respectively) was significantly lower than that for the CTR1 and CTR2 groups (2.76 ± 0.40 g, *p*<0.05 and 3.06 ± 0.14 g, *p*<0.0001, respectively) (Figure 1B). When the two-kidney weight was related to the rat’s body weight, these differences were no longer significant.

The LIS group had a significantly lower (*p*<0.0001) UPE (53.63 ± 34.96 mg·day^-1^) than the CTR1 group (i.e., 367.40 ± 133.60 mg·day^-1^) (85.40% difference) (Figure 1C). The DMTU group also had a significantly lower (*p*<0.05) UPE (30.00 ± 19.00 mg·day^-1^) compared to the CTR2 group (168.00 ± 80.29 mg·day^-1^) (82.10% difference). The significant difference between the CTR1 and the CTR2 groups resulted from the significant difference in age between the two groups (28.80 ± 2.72 and 18.62 ± 0.67 weeks, respectively), which is consistent with previous observations that the progress of renal pathology in male MWF rats exacerbates with age (33). UPE levels did not differ between the LIS group and the DMTU group.

The SBP was significantly lower for the LIS and DMTU groups (117.25 ± 6.98 and 136.92 ± 6.33 mmHg, respectively) than for the CTR1 and CTR2 groups (151.00 ± 20.14, *p*<0.0001 and 165.75 ± 4.60, *p*<0.02) (Figure 1D). Both lisinopril and dimethylthiouria lowered the SBP levels to normal values, which were significantly lower for the LIS group (*p*<0.05) than for the DMTU group; the levels for both groups were comparable to those for healthy Wistar rats (34).

The UPE and SBP levels for the LIS group are consistent with previous observations of the beneficial antiproteinuric and antihypertensive effects of ACE inhibitors in MWF rats (32, 33). Although ACE inhibits the degradation of bradykinin and fosters its hypotensive action, the principal pharmacological and clinical effects of ACE inhibitors were assumed to arise from the suppression of the synthesis of angiotensin II (35) and from the reduction of ROS (36). The antihypertensive effect of ACE inhibitors was accompanied by decreases in both oxidative stress and vascular hypertrophy, which implies that these processes are redox-sensitive in stroke-prone spontaneously hypertensive rats (37). Moreover, ACE inhibitors have been shown to inhibit vascular remodeling and to reduce ROS in these rats by reducing NAD(P)H oxidase and upregulating Cu/Zn superoxide dismutase (36).

The UPE for the DMTU group is also consistent with observations of treatment with the ROS scavenger DMTU, which has been shown to significantly attenuate anti-GBM antibody-induced proteinuria (2). DMTU treatment has also been shown to reduce proteinuria in various models of glomerular injury (22, 38). The protective effects of DMTU in neutrophil-dependent injuries has been shown to derive from scavenging either the hydroxyl radical or hypochlorous acid (2, 8, 39).

### GBM and Albumin Physical Characteristics

No significant differences were observed among the different groups for GBM film thickness (δ_bm_), implying that the consolidation process resulted in homogenous and reproducible GBM layers for all study groups (Table 1). The average σbm (5.57 ± 0.45 × 10^-4^ cm) was used for subsequent calculations. Albumin diffusivity (D) was estimated using the Stokes-Einstein relation [Eq. 8] for albumin solutions (4 g·dL^-1^) at 25°C, which was calculated as 5.76 ± 0.11 × 10^-7^(cm^2^·sec^-1^). Based on the average values for D and δ_bm_, an average Ps value of 10.81 ± 0.87 × 10^-4^ (cm·sec^-1^) was calculated for albumin solutions [Eq. 9].

**Table 1:** Morphometric measurements for the glomerular basement membrane (GBM) and physical characteristics of albumin based on the results from an ultrafiltration cell for all groups of experimental rats

	LIS	CTR1	DMTU	CTR2

Number of Rats	8	7	8	7
Age (*weak*)	27.15 ± 1.10	28.80 ± 2.72	17.71 ± 0.52	18.62 ± 0.67
GBM Thickness, δ_bm_ × 10^-4^ (*cm*)	5.60 ± 1.25	6.16 ± 0.95	5.08 ± 1.60	5.43 ± 1.31
Albumin Diffusivity, D × 10^-7^ (*cm* ^2^·*sec*^-1^)	5.81 ± 0.19	5.82 ± 0.11	5.60 ± 0.17	5.82 ± 0.02
Albumin Permeability, P_s_ × 10^-4^ (*cm·sec*^-1^)	10.81 ± 2.42	9.63 ± 1.52	11.70 ± 3.23	11.11 ± 2.45
Albumin concentration difference, ΔC (*g·dL*^-1^)				
50 mmHg	4.54 ± 0.52	4.59 ± 1.15	6.52 ± 0.87	5.67 ± 0.17
100 mmHg	5.23 ± 0.60^a^	5.02 ± 1.51	6.77 ± 0.42	5.43 ± 0.26
200 mmHg	5.21 ± 0.53^a^	5.15 ± 0.96^a^	7.26 ± 0.77^a^,^b^	6.52 ± 0.46^a^,^b^
300 mmHg	4.96 ± 0.64	5.51 ± 1.14^a^,^b^	6.82 ± 1.02^a^	6.07 ± 0.36^a^,^b^

LIS, Lisinopril treated rat group; CTR1, first control rat group; DMTU, dimethylthiouria treated rat group; CTR2, second control rat group. Values are expressed as mean ± SD.

a *p*<0.05 vs. 50 mmHg;

b *p*<0.05 vs. 100 mmHg.

### Solvent and Solute Fluxes

**Figure 2:**
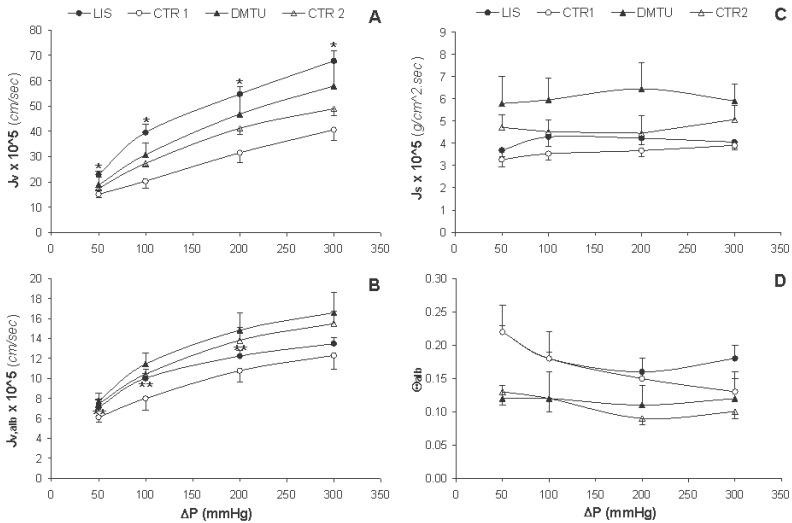
Tracks of change of the volumetric solvent flux in the absence of albumin (J_v_) and presence of albumin (J_v,alb_) at a concentration of 4g.dL^-1^, albumin flux (J_s_), and albumin fractional clearance (Θ_alb_) with the hydrostatic pressure difference (ΔP) for lisinopril treated rats (LIS, n=8), its negative control group (CTR1, n=7), dimethylthiouria treated rats (DMTU, n=8), and its negative control group (CTR2, n=7). Data are presented as mean ±SD. *p<0.02 vs. CTR1 and **p<0.05 vs. CTR1.

Figures 2A and 2B show the solvent flux in the absence and in the presence of albumin (J_v_ and J_v,alb_, respectively) for all groups of experimental animals. As ΔP increased, J_v_ and J_v,alb_ increased in an identical linear fashion; nonetheless, the J_v_ values were approximately 2.5 to 5 times higher than the J_v,alb_ values at the same ΔP. For the LIS group, J_v_ was significantly higher (*p*<0.02) compared to that of the CTR1 group at all ΔP; however, the J_v_ values for the DMTU group did not significantly differ when compared to those of the CTR2 group (Figure 2A). The J_v,alb_ values for the LIS group at ΔP 50, 100, and 200 were significantly higher (*p*<0.05) than those for the CTR1 group but the J_v,alb_ values for the DMTU group at all ΔP were not significantly different from those for the CTR2 group (Figure 2B). Albumin flux (J_s_) and albumin fractional clearance (Θ_alb_) maintained nearly stable with increasing ΔP within the same group and without any significant differences among groups (Figures 2C and 2D).

The Θalb *in vitro* (14.56 ± 0.04) was much higher than that *in vivo* for the intact glomerulus by micropuncture techniques in the experimental rat model (i.e., ~3 × 10^-4^) (40). This huge difference was assumed to have derived from the absence of endothelial and epithelial layers in GBM preparations (41); moreover, loss of GBM proteins and modulation of the GBM-protein structure are also considered critical for the high degree of restriction of plasma proteins across the intact glomerulus (6). Although albumin is negatively charged at a pH7.4, it showed no significant changes in behavior when filtered at a pH of 5.7 (34); thus size rather than charge may be the principal determinant of filtration for this large protein.

An ideal ultrafiltration membrane would be expected to show linear increases in J_v,alb_ and J_s_, whereas Θ_alb_ should remain constant with increasing ΔP, provided that J_s_ is dominated by convection [as explained below after Eq. 15]. In our study, the behavior of the basement membrane film deviated from this ideal situation for Js and Θ_alb_ (Figure 2C and D), which has been previously explained as a consequence of the compression of GBM films under the initial packing pressure (27, 42).

### Effect of Hydrostatic Pressure and Albumin Concentration on Hydraulic Permeability

**Figure 3:**
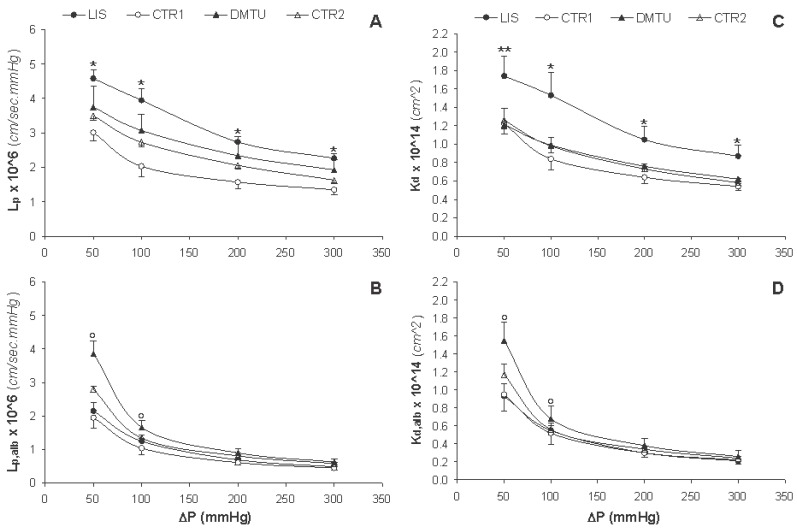
Changes in actual hydraulic and local Darcy permeabilities in absence of albumin (L_p_ and K_d_, respectively) and presence of albumin (L_p,alb_ and K_d,alb_, respectively) associated with increment changes in the hydrostatic pressure difference (ΔP) for lisinopril treated rats (LIS, n=8), its negative control group (CTR1, n=7), dimethylthiouria treated rats (DMTU, n=8), and its negative control group (CTR2, n=7). Data are presented as mean ± SD. *p<0.02 vs. CTR1, **p<0.05 vs. CTR1, and °*p*<0.05 vs. CTR2.

The hydraulic permeability in the absence of albumin (L_p_) decreased linearly with increasing ΔP for all study groups (Figure 3A). The L_p_ values for the LIS group were significantly higher (*p*<0.02) than those for the CTR1 group at all ΔP; however, the L_p_ values for the DMTU group were not significantly different from those for the CTR2 group. The hydraulic permeability of albumin solutions (L_p,alb_) also decreased by increasing ΔP for all groups (Figure 3B), yet in a curvilinear fashion, reaching lower values in comparison with L_p_. The actual hydraulic permeability values (L_p,alb_, Table 2), which were calculated using Θ_alb_ = C_f_/C_r_ instead of Θ_alb_, given by Eq. 6, were lower than the L_p,alb_ values yet showed the same inverse relationship with ΔP. The L_p,alb_ and L’_p,alb_ values at 100, 200 and 300 mmHg were significantly lower (*p*<0.05) than those at 50 mmHg for all study groups (Table 2), with the values at 200 and 300 mmHg being significantly lower (*p*<0.05) than those at 100 mmHg. The L_p,alb_ values for the LIS group were not significantly different from those for the CTR1 group at all ΔP; however, the L_p,alb_ values for the DMTU group were significantly higher (*p*<0.05) than those for the CTR2 group at all ΔP.

**Table 2:** Effect of concentration polarization on hydraulic permeability of packed GBM films for albumin solutions (4 g·dL^-1^ concentration)

Group	ΔP	B	L_p,alb_	L_p,alb_
	mmHg		×10^-6^cm·sec^-1^·mmHg^-1^	

**LIS**	50	1.18 ± 0.02	1.91 ± 0.21	2.15 ± 0.25
	100	1.26 ± 0.01*	1.16 ± 0.06*	1.25 ± 0.07*
	200	1.33 ± 0.02*º	0.66 ± 0.03*º	0.69 ± 0.03*º
	300	1.37 ± 0.02*º	0.47 ± 0.02*º	0.48 ± 0.02*º
**CTR1**	50	1.15 ± 0.01	1.72 ± 0.21	1.95 ± 0.30
	100	1.21 ± 0.03*	0.94 ± 0.16*	1.04 ± 0.20*
	200	1.28 ± 0.03*º	0.58 ± 0.07*	0.61 ± 0.07*
	300	1.33 ± 0.04*º	0.43 ± 0.05*º	0.45 ± 0.06*º
**DMTU**	50	1.19 ± 0.02	2.77 ± 0.06	3.86 ± 0.38^a^
	100	1.30 ± 0.03*	1.43 ± 0.14*	1.66 ± 0.21*^a^
	200	1.41 ± 0.06*º	0.82 ± 0.10*º	0.90 ± 0.12*º^a^
	300	1.47 ± 0.07*º	0.59 ± 0.07*º	0.63 ± 0.09*º^a^
**CTR2**	50	1.19 ± 0.01	2.32 ± 0.07	2.79 ± 0.11
	100	1.27 ± 0.02*	1.23 ± 0.06*	1.34 ± 0.08*
	200	1.37 ± 0.04*º	0.76 ± 0.07*º	0.81 ± 0.08*º
	300	1.44 ± 0.04*º	0.56 ± 0.04*º	0.58 ± 0.04*º

ΔP, hydrostatic pressure difference; B, concentration polarization factor; L’_p,alb_ : effective hydraulic permeability; L_p,alb_: actual hydraulic permeability; LIS, Lisinopril treated rat group; CTR1, first control rat group; DMTU, dimethylthiouria treated rat group; CTR2, second control rat group. Values are expressed as mean ± SE.

* *p*<*0.05* vs. 50 mmHg,

º *p*<*0.05* vs. 100 mmHg, and

a *p*<*0.05* vs. CTR1, CTR2, and LIS.

Previous results of *in vivo* studies have shown that the administration of ACE inhibitors have renoprotective effects; decreasing proteinuria and dextran clearance and increasing the hydraulic permeability in the MWF rat model, yet the exact mechanism(s) of these favorable effects is still unclear (33). DMTU treatment have been shown also to be accompanied by reduced albumin clearance and increased hydraulic permeability mediated by a DMTU-induced increase in renal glutathione (22).

According to Eq. 4, if the plot relating J_v,alb_ and ΔP is linear and passes through the origin, then the L_p,alb_ calculated at each ΔP will be identical. An example of this is the case of dialysis membranes when the “distending pressure”, which is equal to the plasma oncotic pressure, is subtracted from the applied pressure (43). However, when this relationship is curvilinear or does not pass through the origin, as was the case in the present study, L_p,alb_ will be different at each pressure level (Figure 2B, 3B). The decrease in L_p,alb_ as ΔP increased has been attributed to the compressibility of the GBM at high and physiologic pressures (42). The ultrafiltration coefficient has also been shown to follow the same profile of dependence on ΔP in many *in vivo* glomerular micropuncture studies in association with a wide range of experimental and pharmacological maneuvers (33, 44-46).

In this study, skeletons of GBM were consolidated under high pressure, so that many layers of GBM comprised the filtration system. If we assume that the GBM thickness is not altered by the consolidation process, then the actual number of GBM layers calculated on the basis of the mean thickness of the GBM film (5.57 ± 0.45 × 10^-4^ cm) and the mean thickness of the GBM from comparable rats (230 × 10^-7^ cm) (32) is approximately 25. The GBM layers can be considered as resistors in series (27); thus the hydraulic permeability for a single GBM layer is approximately 25 times the measured L_p,alb_ value in the present experiments. To correct L_p,alb_ experimental values for the difference between room temperature and body temperature, we introduced the ratio of the viscosity of water at 25°C to that at 37°C (i.e., 1.3), thus we estimate that

[Eq. 13] $$L_{p,\mathrm{alb}}=25\times 1.3\times 1.95\times 10^{-6}=0.63\times 10^{-4}\left({\mathrm{cm}\cdot \sec}^{-1}\cdot {\mathrm{mmHg}}^{-1}\right)$$

for one GBM layer at ΔP = 50 mmHg, 4 g·dL^-1^ albumin concentration, and 37°C of CTR1 rats, which is approximately 2 times higher than typical micropuncture estimates of L_p,alb_ in the intact rat glomerulus (47). Other research groups have estimated L_p,alb_ values that were five times higher than micropuncture estimates (27).

During the filtration process, the movement of albumin across the semi-permeable GBM film results from diffusion, and from convection, where momentum is transferred from the solvent to albumin molecules (42). The relative magnitude of these two processes is expressed by the transmembrane Péclet number (P_e_), which is given by

[Eq. 14] $$P_{e}=J_{v,\mathrm{alb}}\left({1-\sigma }_{\mathrm{alb}}\right)/P_{s}$$

where J_v,alb_ is the solvent volume flux (Eq. 3), Θ_alb_ is the albumin reflection coefficient, and Ps is the albumin permeability coefficient (Eq. 9). P_e_ values were calculated to have an average value of 0.02 for all GBM membranes at all ΔP. Thus, albumin flux (J_s_, given by Eq. 10) as a result of the two concurrent mechanisms convection and diffusion is given by (26):

[Eq. 15] $$J_{e}=J_{v,\mathrm{alb}}\left({1-\sigma }_{\mathrm{alb}}\right)C_{m}+P_{s}\left(C_{m}-C_{f}\right)P_{e}/\left({\mathrm{ExpP}}_{e}-1\right)\left({g\cdot \mathrm{cm}}^{-2}\cdot \sec^{-1}\right)$$

where C_m_ is the albumin concentration in the overstanding solution and C_f_ is the albumin concentration in the filtrate, noting that the difference of albumin concentration on both sides of the GBM film (i.e., ΔC = C_m_ - C_f_) tended to increase by increasing ΔP. The ΔC values at ΔP 200 and 300 mmHg were significantly higher than those at 50 and 100 mmHg for all study groups (Table 1).

When albumin is filtered through a partially obstructive membrane, the rejected macromolecules tend to accumulate at the membrane surface, which is known as “concentration polarization” (B, given by Eq. 7) (42). The effects of B become more obvious as the filtration pressure is increased (Table 2), since the increased buffer velocity towards the membrane tends to sweep more macromolecules to the membrane surface. This effect has two consequences, the first is to increase the local albumin concentration (C_m_), which in turn increases the osmotic potential across the membrane (Π_alb_) opposing the applied hydrostatic pressure (ΔP) reducing the solvent flux (J_v,alb_, Eq. 4). Thus by increasing filtration ΔP, J_v,alb_ tends to fall below the values observed when buffer is filtered alone, J_v_ (Figure 2A and 2B). The second effect is to increase the solute flux across the membrane (J_s_), since it is determined by the albumin concentration at the membrane surface (C_m_) rather than that in the bulk solution (C_r_) (Figure 2C).

### Darcy Permeability

The Darcy permeability is a specific measure of fluid permeability, which is independent from the GBM thickness and fluid viscosity. The Darcy permeability values in the absence and presence of albumin (K_d_ and K_d,alb_, respectively) are summarized in Table 3. Both K_d_ and K_d,alb_ values at 100, 200 and 300 mmHg were significantly lower (*p*<0.05) than those at 50 mmHg for all study groups. The K_d_ values for the LIS group were also significantly higher (*p*<0.05) than those for the CTR1 group at all ΔP, whereas the K_d_ values for the DMTU group were comparable to those for the CTR2 group (Figure 3C). Figure 3D shows the rapid progressive decline of K_d,alb_ by increasing ΔP in the presence of albumin for all study groups in comparison with K_d_. The K_d,alb_ values for the LIS group were not significantly different from those for the CTR1 group at all ΔP yet, the K_d,alb_ values for the DMTU group were significantly higher (*p*<0.05) than those for the CTR2 group only at 50 and 100 mmHg.

**Table 3:** Effect of transmembrane hydrostatic pressure on local Darcy permeability of packed GBM films in the absence and presence of albumin (4 g·dL^-1^ concentration)

Group	ΔP mmHg	K_d_	K_d,alb_

		×10^-14^ cm^2^	

**LIS**	50	1.74 ± 0.22^a^	0.93 ± 0.14
	100	1.53 ± 0.25^b^	0.55 ± 0.05*
	200	1.05 ± 0.14*º^b^	0.30 ± 0.02*º
	300	0.87 ± 0.12*º^b^	0.21 ± 0.02*º
**CTR1**	50	1.22 ± 0.05	0.95 ± 0.18
	100	0.84 ± 0.12*	0.52 ± 0.13*
	200	0.64 ± 0.07*	0.30 ± 0.05*º
	300	0.54 ± 0.04*º	0.22 ± 0.04*º
**DMTU**	50	1.21 ± 0.10	1.55 ± 0.20^c^
	100	0.99 ± 0.08	0.68 ± 0.14*^c^
	200	0.76 ± 0.06*	0.38 ± 0.08*º
	300	0.62 ± 0.05*º	0.26 ± 0.07*º
**CTR2**	50	1.26 ± 0.13	1.17 ± 0.12
	100	0.98 ± 0.09*	0.56 ± 0.07*
	200	0.73 ± 0.06*º	0.34 ± 0.02*º
	300	0.58 ± 0.05*º	0.24 ± 0.02*º

ΔP, hydrostatic pressure difference; K_d_: local Darcy permeability in absence of albumin; K_d,alb_: actual Darcy permeability in presence of albumin; LIS, Lisinopril treated rat group; CTR1, first control rat group; DMTU, dimethylthiouria treated rat group; CTR2, second control rat group. Values are expressed as mean ± SE.

* *p*<*0.05* vs. 50 mmHg,

º *p*<*0.05* vs. 100 mmHg.

a *p*<*0.05* vs. CTR1,

b *p*<*0.02* vs. CTR1,

c *p*<*0.05* vs. CTR2.

### CONCLUSIONS

We studied the permeability properties of bare isolated GBM to water and albumin in the MWF experimental rat model, which develops spontaneously proteinuria by age. Two parallel pharmacological regimens (i.e., ACE inhibitors and ROS scavengers) were used to treat proteinuria in these animals. Ultrafiltration experiments showed that the GBM exhibits high water permeability and restricts the passage of macromolecules such as albumin, although to a much lesser extent than the intact glomerulus. We attributed these differences to the absence of endothelial and epithelial layers in GBM preparations; and possibly to the loss or modulation of the GBM-protein structure, which may occur during the GBM isolation procedure.

It has been shown that increased intra-renal oxidant stress, owing to an overproduction of ROS and dysregulated tubular antioxidant enzymes, can induce overexpression of fibrogenic cytokines and chemoattractants, as well as increased transcription and synthesis of extracellular matrix proteins, leading to tubular loss and fibrogenesis (19). Both animals treated with ACE inhibitors and ROS scavengers had higher hydraulic and Darcy permeability, either in the absence or presence of albumin, in comparison with control animals; suggesting their direct effect on GBM structure. A possible explanation of these favorable effects maybe attributed to maintaining the GBM matrix structure by reducing its integral proteins degradation (i.e., entactin and laminin) and cross-linking (i.e., type IV collagen) (8). This hypothesis is in line with previous studies showing that treatment with ACE inhibitors, which are also ROS scavengers, was accompanied by decreases of both oxidative stress and vascular hypertrophy, inhibition of vascular remodeling, and reduction of ROS; due to the reduction of NAD(P)H oxidase and the upregulation of Cu/Zn superoxide dismutase (36, 37). Filtration experiments showed also that size, rather than charge, maybe the principal determinant for filtration of macromolecules across the GBM and that the GBM is the principle barrier responsible for permeability properties of the GCW.
